# Supplementary material for: The Structural Integrity of the Model Lipid Membrane during Induced Lipid Peroxidation: The Role of Flavonols in the Inhibition of Lipid Peroxidation
Source: Antioxidants (Basel). 2020 May 15;9(5):430. doi: 10.3390/antiox9050430 (PMC7278707; doi:10.3390/antiox9050430)
Supplement: Supplementary file 1 [file antioxidants-09-00430-s001.pdf]

The structural integrity of the model lipid membrane during induced lipid peroxidation: the role of flavonols in the inhibition of lipid peroxidation

Anja Sadžak [1], Janez Mravljak [2], Nadica Maltar-Strmečki [1], Zoran Arsov [3], Goran Baranović [1], Ina Erceg [1], Manfred Kriechbaum [4], Vida Strasser [1], Jan Přibyl [5] and Suzana Šegota [1,*]

[1] Ruđer Bošković Institute, Zagreb, Croatia
[2] Faculty of Pharmacy, University of Ljubljana, Ljubljana, Slovenia
[3] Jožef Stefan Institute, Ljubljana, Slovenia
[4] Institute of Inorganic Chemistry, Graz University of Technology, Graz, Austria
[5] CEITEC, Masaryk University, Brno, Czech Republic
* Correspondence: suzana.segota@irb.hr

Table S1. Derivatized Oxo LPP identified after peroxidation of DOPC liposomes with and without inserted flavonols.

Name	Structure	Monoisotopic mass	*m/z* after derivatis.	DOPC + QUER Delta ppm				DOPC + MCE Delta ppm			DOPC+MCI Delta ppm		
				$x = 0$	$x = 0.01$	$x = 0.05$	$x = 0.10$	$x = 0.01$	$x = 0.05$	$x = 0.10$	$x = 0.01$	$x = 0.05$	$x = 0.10$
Methanal	CH_2O	30.011	288.13	288.13416<br>-0.375	–	–	288.13383<br>-1.520	–	288.13391<br>-1.242	288.13370<br>-1.971	288.13365<br>-2.145	288.13368<br>-2.041	–
Acrolein	C_3H_4O	56.026	314.15	314.14902<br>-2.859	–	–	–	–	–	314.149<br>-2.381	–	–	–
Butanal	C_4H_8O	72.058	330.18	330.18094<br>-0.842	330.18029<br>-2.811	330.18051<br>-2.145	330.18036<br>-2.599	–	330.18054<br>-2.054	330.18048<br>-2.236	–	330.18039<br>-2.508	330.18057<br>-1.963
Hexanal	$C_6H_{12}O$	100.089	380.19	358.21222<br>-0.833	358.21188<br>-1.782	358.21154<br>-2.731	358.21216<br>-1.000	358.21194<br>-1.614	358.21198<br>-1.503	358.21194<br>-1.614	–	358.21201<br>-1,419	–
6-Hydroxy-heptanal	$C_7H_{14}O_2$	130.099	388.22	388.22403<br>2.440	388.22342<br>0.868	–	–	–	–	–	–	–	–
Hydroxy-octenal	$C_8H_{14}O_2$	142.099	400.22	400.22293<br>-0.382	400.22168<br>-3.505	–	400.22330<br>0.542	–	400.22221<br>-2.181	–	–	400.22197<br>-2.781	–
Hydroxy-oxo-undecenoic acid[a]	$C_{11}H_{18}O_4$	214.120	472.24	473.25073<br>-2.762<br>471.23810<br>3.635	–	–	–	–	–	–	–	–	473.25259<br>1.168
Oxo-decenedioic acid[a]	$C_{10}H_{14}O_5$	214.084	472.21	473.21609<br>0.863	473.21539<br>-0.553	–	–	473.21530<br>-0.743	–	–	–	–	–
Oxo-tridecatrienoic acid[a]	$C_{13}H_{18}O_3$	222.126	480.25	479.24329<br>3.792	481.25909<br>4.088	–	–	481.25897<br>3.839	–	–	–	–	–
Oxo-tridecadienoic acid[a]	$C_{13}H_{20}O_3$	224.141	482.26	481.25778<br>1.366		482.26508<br>0.585	–		–	–	–	–	–
Oxo-dodeca-dienedioic acid[a]	$C_{12}H_{16}O_5$	240.100	498.23	499.23343<br>4.263	–	499.23181<br>1.018	–	–	–	–	–	499.23126<br>-0.084	499.23273<br>2.861
Oxo-dodecenedioic acid[a]	$C_{12}H_{18}O_5$	242.115	500.24		500.23923<br>0.206		–	500.23956<br>0.866	–	–	–		

[a] For these compounds, m/z values that correspond to protonated or deprotonated species were observed, and assigned if possible.

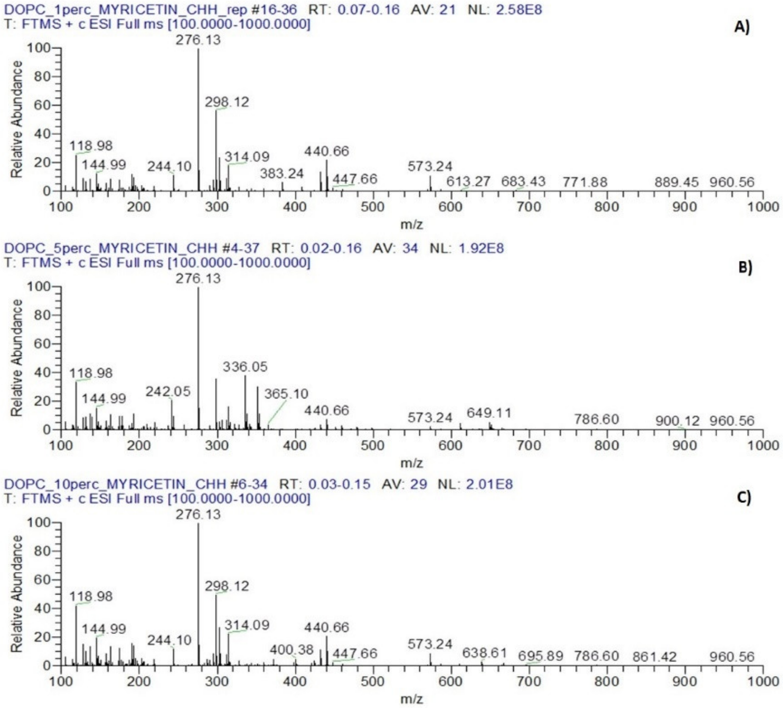

Figure S1**.** Mass spectrum of LPP ions of DOPC with (A) x_{MCE} = 0.01, (B) x_{MCE} = 0.05 and (C) x_{MCE} = 0.10.

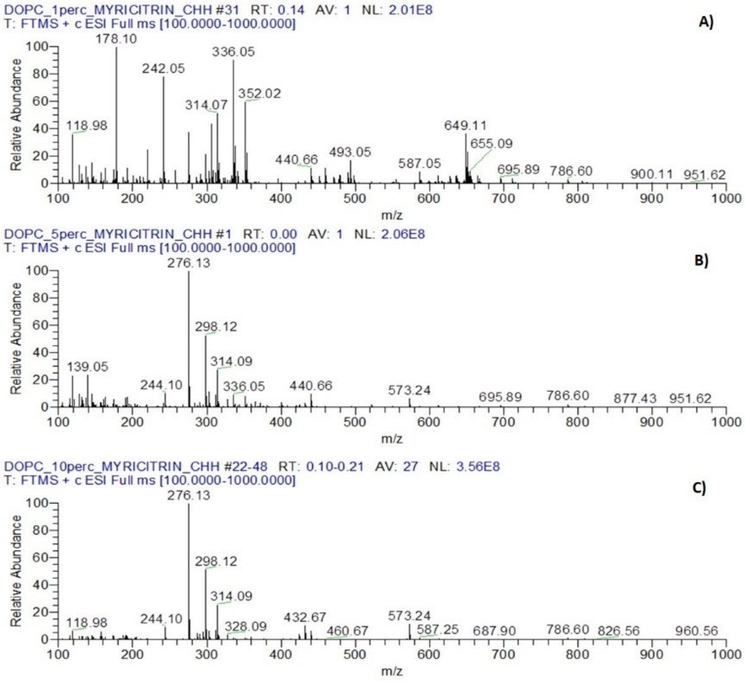

Figure S2**.** Mass spectrum of LPP ions of DOPC with (A) x_{MCI} =0.01, (B) x_{MCI} =0.05 and (C) x_{MCI} =0.10.

Table S2. The inhibition of lipid peroxidation for three molar fractions of flavonols.

x	R_i		
	**QUER**	**MCE**	**MCI**
0.01	-0.69±0.46	-0.91±0.58	-1.03±0.42
0.05	-1.54±0.49	-1.02±0.58	-1.10±0.43
0.10	-1.70±0.53	-0.93±0.58	-1.74±0.54

Table S3. Nanostructures of the DOPC lipid assigned as m: multilamellar, b: single bilayer and b+: bilayer plus traces of multilayers.

**Sample**	**Nanostructure**
DOPC	-
DOPC/ H_2O_2 + Fe^{2+}	m
DOPC/QUER (x = 0.01)	m
DOPC/QUER (x = 0.01) / H_2O_2 + Fe^{2+}	b+
DOPC/QUER (x = 0.05)	m
DOPC/QUER (x = 0.05) / H_2O_2 + Fe^{2+}	b+
DOPC/QUER (x = 0.1)	m
DOPC/QUER (x = 0.1) / H_2O_2 +Fe^{2+}	-
DOPC/MCE (x = 0.01)	m
DOPC/MCE (x = 0.01)/H_2O_2 +Fe^{2+}	b+
DOPC/MCE (x = 0.05)	b
DOPC/MCE (x = 0.05)/H_2O_2+ Fe^{2+}	m
DOPC/MCE (x = 0.1)	m
DOPC/MCE (x = 0.1)/ H_2O_2 +Fe^{2+}	b
DOPC/MCI (x = 0.01)	m
DOPC/MCI (x = 0.01)/H_2O_2 +Fe^{2+}	b
DOPC/MCI (x = 0.05)	m
DOPC/MCI (x = 0.05)/H_2O_2 +Fe^{2+}	b+
DOPC/MCI (x = 0.1)	m
DOPC/MCI (x = 0.1)/ H_2O_2 +Fe^{2+}	b+

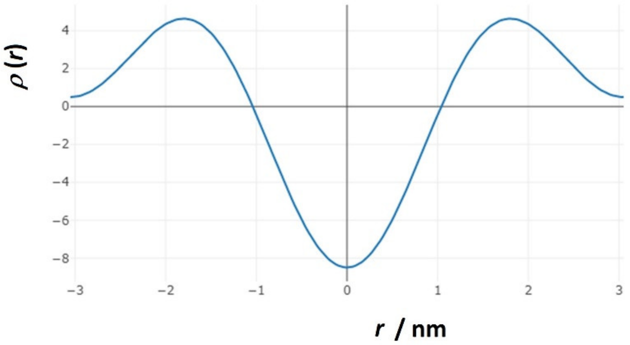

Figure S3. 1D electron density profile across the DOPC bilayer with inserted MCE and interstitial water-layer of one unit in the multilayer lipid system (x = 0.1). The dimension of one symmetric unit is r = 6.1 nm. The electron density is lowest at the position of the methylgroups of the lipid (r = 0) and highest at the position of the phosphatidyl headgroups of the lipid (r = ± 1.8 nm). The center of the interstitial water layer is located at r = ± 3.05 nm.

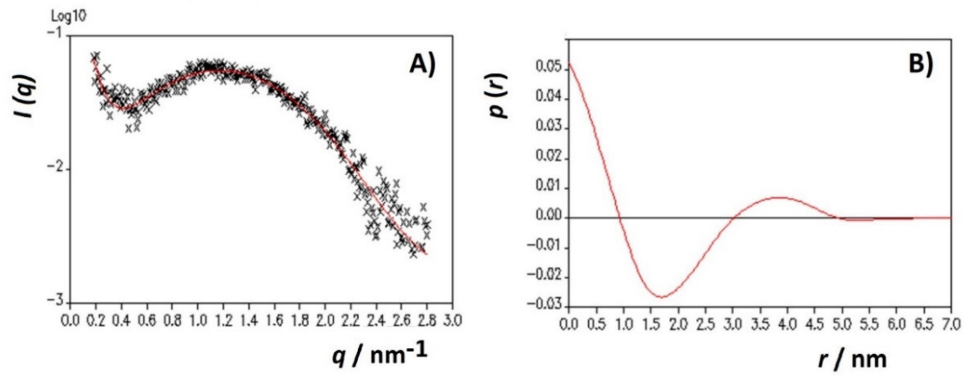

Figure S4. A) Experimental SAXS-data (black x) and fit (red line) and B: calculated $p(r)$-function calculated up to r = 7 nm. Calculation of the $p(r)$-function from the SAXS curve $I(q)$ of DOPC-MCI_LP (x = 0.1) using the program GIFT assuming lamellar symmetry (B).

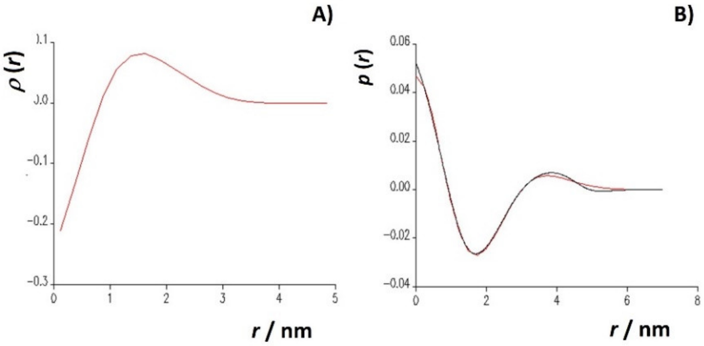

Figure S5. Calculation of the electron density function from the $p(r)$-function of DOPC-MCI_LP (x = 0.1) using the software DECON assuming lamellar symmetry, electron-density $\rho(r)$ calculated up to r = 5 nm (one symmetric half of the lamellar bilayer (A), and the fit of the $p(r)$-function (red) to the one obtained by GIFT (black, see Fig S4(B)) (B).

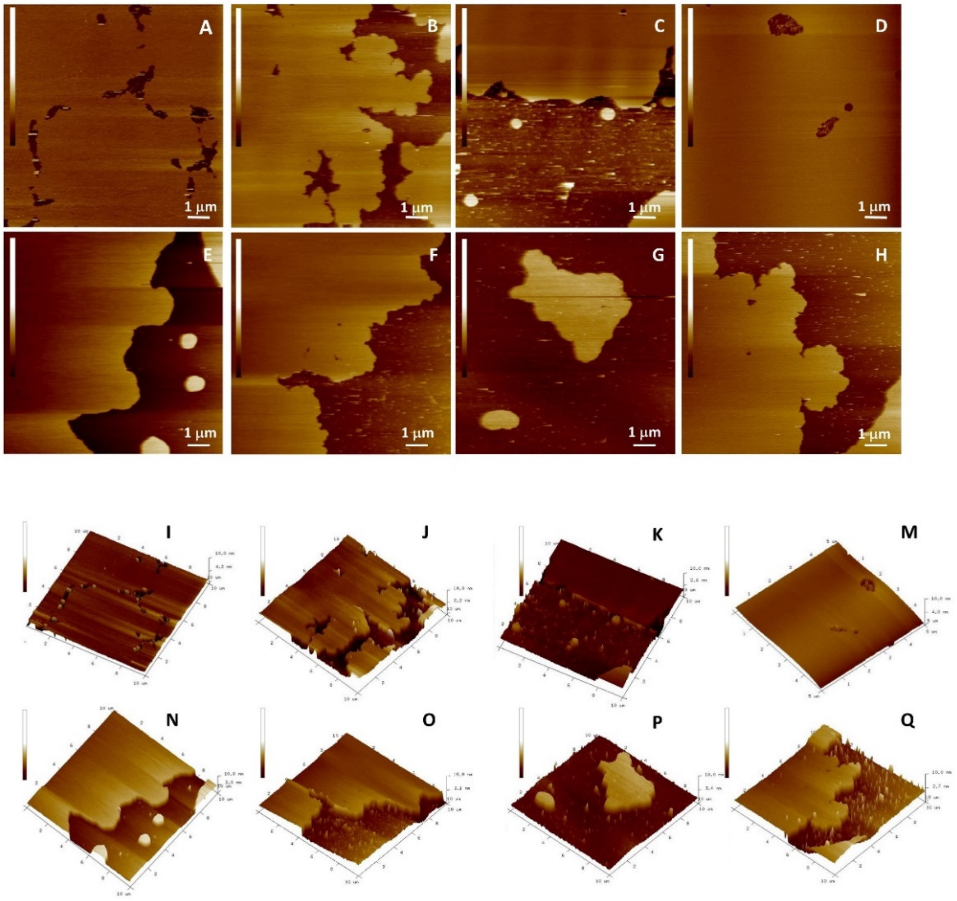

Figure S6. Top view of height AFM images on the model (DOPC) SLB without (x=0) and with inserted myricitrin (MCI) at different molar fraction before induced lipid peroxidation: 2D view: (A) DOPC (x = 0), (B) (x = 0.01), (C) (x = 0.05) and (D) (x = 0.10) and 3D view: (I) DOPC (x = 0) (J) (x = 0.01), (K) (x = 0.05) and (M) (x = 0.10). After induced lipid peroxidation 2D view: (E) DOPC (x = 0), (F) (x = 0.01), (G) (x = 0.05) and (H) (x = 0.10); 3D view (N) DOPC (x = 0); (O) (x = 0.01), (P) (x = 0.05) and (Q) (x = 0.10). Scales of all images are 6 nm.

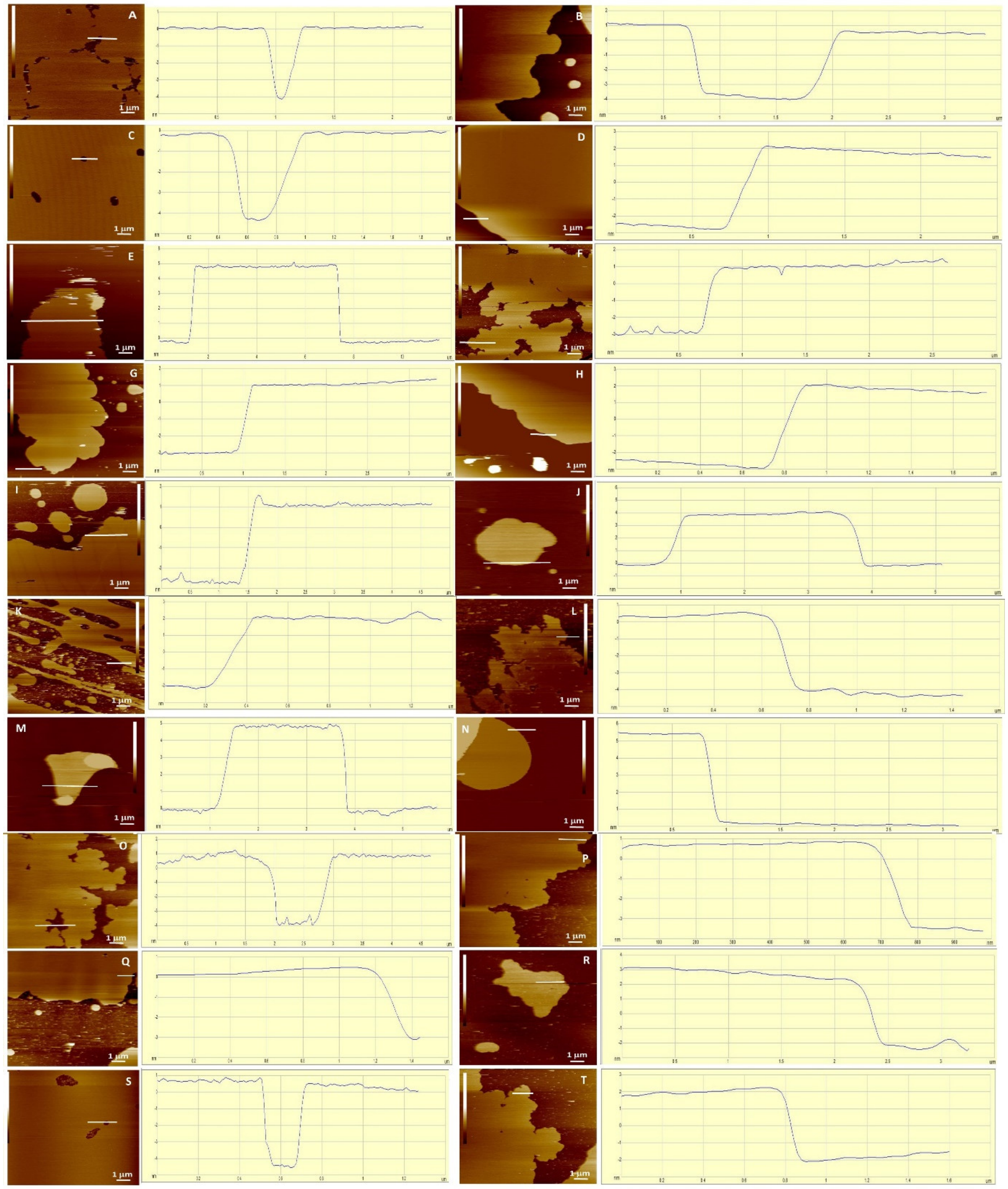

Figure S7. 2D-height image and cross sections of the model (DOPC) SLB without and with inserted flavonols at different molar fraction before induced lipid peroxidation: (A) DOPC (x = 0), (C) (x_{QUE} = 0.01), (E) (x_{QUE} = 0.05), (G) (x_{QUE} = 0.10), (I) (x_{MCE} = 0.01), (K) (x_{MCE} = 0.05), (M) (x_{MCE} = 0.10), (O) (x_{MCI} = 0.01), (Q) (x_{MCI} = 0.05) and (S) (x_{MCI} = 0.10), and after lipid peroxidation (B) DOPC (x = 0), (D) (x_{QUE} = 0.01), (F) (x_{QUE} = 0.05), (H) (x_{QUE} = 0.10), (J) (x_{MCE} = 0.01), (L) (x_{MCE} = 0.05), (N) (x_{MCE} = 0.10), (P) (x_{MCI} = 0.01), (R) (x_{MCI} = 0.05) and (T) (x_{MCI} = 0.10). Scales of all images are 6 nm.

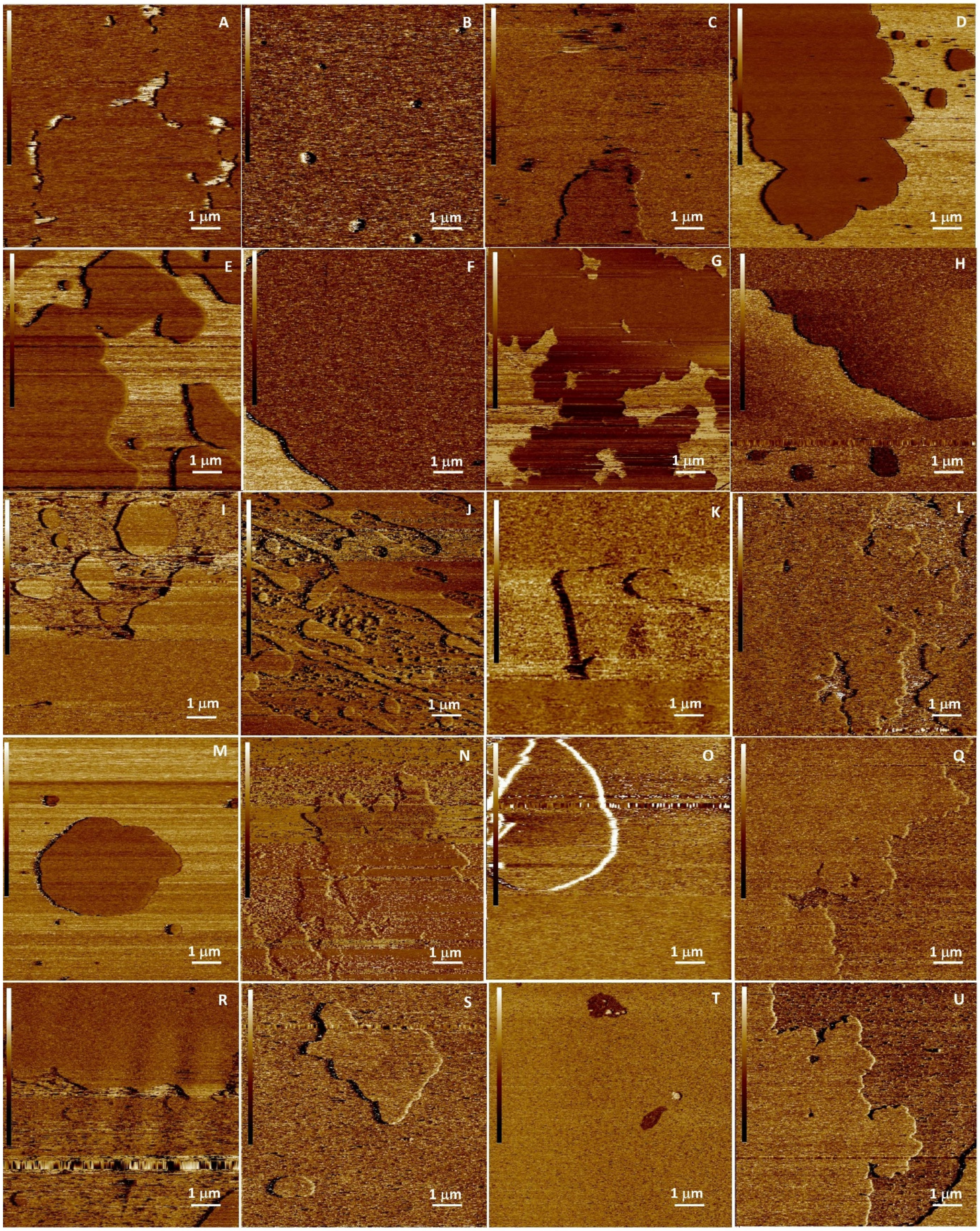

Figure S8. Nanomechanical maps of the model (DOPC) SLB without and with inserted flavonols at different molar fraction before induced lipid peroxidation (A) DOPC (x = 0), (B) (x_{QUE} = 0.01), (C) (x_{QUE} = 0.05), (D) (x_{QUE} = 0.10), (I) (x_{MCE} = 0.01), (J) (x_{MCE} = 0.05), (K) (x_{MCE}=0.10), (L) (x_{MCI} = 0.01), (R) (x_{MCI} = 0.05) and (T) (x_{MCI} = 0.10), and after lipid peroxidation (E) DOPC (x = 0), (F) (x_{QUE} = 0.01), (G) (x_{QUE} = 0.05), (H) (x_{QUE} = 0.10), (M) (x_{MCE} = 0.01), (N) (x_{MCE} = 0.05), (O) (x_{MCE} = 0.10), (Q) (x_{MCI} = 0.01), (S) (x_{MCI} = 0.05) and (U) (x_{MCI} = 0.10). Scales of all images are 40 MPa.

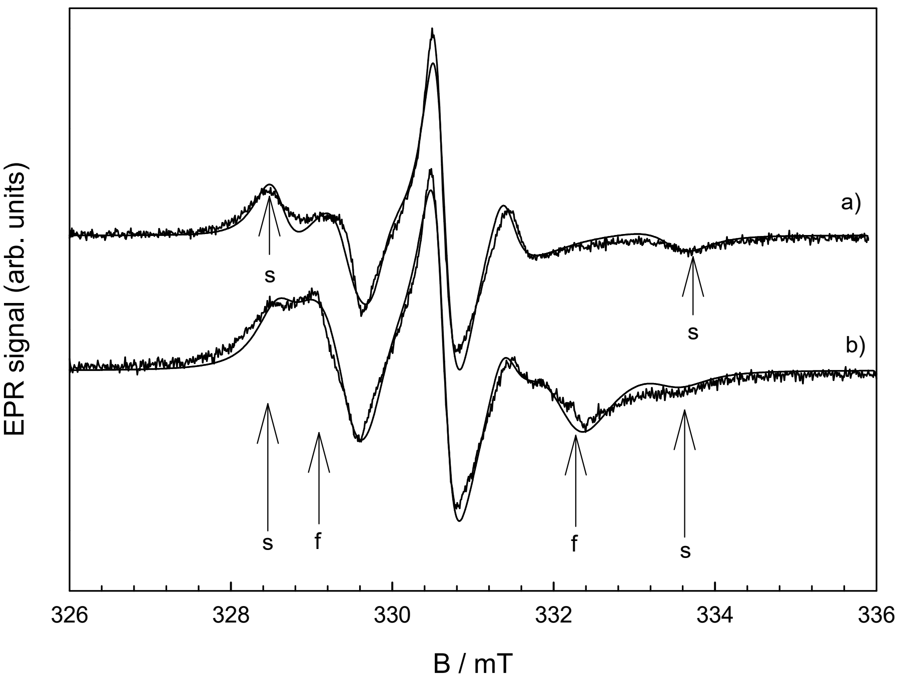

Figure S9. EPR spectra of the control sample (DOPC liposomes) a) before and b) after lipid peroxidation.

Appendix A

The absorbance of the methyl band at 2959 cm^{-1} is the sum of DOPC and lipid peroxidation products' (LPPs) absorbances. The number of methyl groups in the sample is considered constant. Therefore, before and after lipid peroxidation we have:

$$A^0(\nu_{\mathrm{as}}(\mathrm{CH_3})) = \varepsilon_\mathrm{M}(\mathrm{DOPC}) \cdot n_M^0(\mathrm{DOPC}) + \varepsilon_\mathrm{M}(\mathrm{LPP}) \cdot n_M^0(\mathrm{LPP}) \quad \text{(A1)}$$

$$A^{LP}\big(\nu_{as}(\mathrm{CH_3})\big) = \varepsilon_M(\mathrm{DOPC}) \cdot n_M^{LP}(\mathrm{DOPC}) + \varepsilon_M(\mathrm{LPP}) \cdot n_M^{LP}(\mathrm{LPP}), \quad \text{(A2)}$$

where n_M(DOPC) and n_M(LPP) correspond to the number of methyl groups in DOPC and LPPs, respectively, while superscript 0 and LP designate these numbers before and after lipid peroxidation. It will be assumed that the absorption coefficients of the methyl groups are equal, $\varepsilon_\mathrm{M}(\mathrm{DOPC}) = \varepsilon_\mathrm{M}(\mathrm{LPP}) = \varepsilon_\mathrm{M}$. Since the lipid can be oxidized in air (carbonyl compounds were observed in MS of DOPC samples without flavonoids and before LP), it is not correct to presume that $n_\mathrm{M}^0(\mathrm{LPP}) = 0$. The total number of methyl groups is constant, because they are neither formed nor destroyed during lipid peroxidation:

$$n_\mathrm{M}^\mathrm{LP}(\mathrm{DOPC}) + n_\mathrm{M}^\mathrm{LP}(\mathrm{LPP}) = n_\mathrm{M}^0(\mathrm{DOPC}) + n_\mathrm{M}^0(\mathrm{LPP}) = n_\mathrm{M,tot} \quad \text{(A3)}$$

Thus:

$$A^0\big(\nu_\mathrm{as}(\mathrm{CH_3})\big) = \varepsilon_\mathrm{M} \cdot \big(n_\mathrm{M}^0(\mathrm{DOPC}) + n_\mathrm{M}^0(\mathrm{LPP})\big) = \varepsilon_\mathrm{M} \cdot n_\mathrm{M,tot} \quad \text{(A4)}$$

$$A^\mathrm{LP}\big(\nu_\mathrm{as}(\mathrm{CH_3})\big) = \varepsilon_\mathrm{M} \cdot \Big(n_\mathrm{M}^\mathrm{LP}(\mathrm{DOPC}) + n_\mathrm{M}^\mathrm{LP}(\mathrm{LPP})\Big) = A^0(\nu_\mathrm{as}(\mathrm{CH_3})) \quad \text{(A5)}$$

The analogous equations can be written for the carbonyl stretching band

$$A^0(\nu(\mathrm{C{=}O})) = \varepsilon_\mathrm{C}^0(\mathrm{DOPC}) \cdot n_\mathrm{C}^0(\mathrm{DOPC}) + \varepsilon_\mathrm{C}^0(\mathrm{LPP}) \cdot n_\mathrm{C}^0(\mathrm{LPP}) \quad \text{(A6)}$$

$$A^\mathrm{LP}\big(\nu(\mathrm{C{=}O})\big) = \varepsilon_\mathrm{C}^\mathrm{LP}(\mathrm{DOPC}) \cdot n_\mathrm{C}^\mathrm{LP}(\mathrm{DOPC}) + \varepsilon_\mathrm{C}^\mathrm{LP}(\mathrm{LPP}) \cdot n_\mathrm{C}^\mathrm{LP}(\mathrm{LPP}), \quad \text{(A7)}$$

It is known that LPPs are formed from the DOPC radical. From the results of the antioxidative activity measurements of flavonoids, we can assume that the principal mechanism of inhibition involves removal of DOPC radical or the decrease in the rate of its formation, while the rates of parallel reactions that lead from DOPC radical to the final LPPs remain approximately constant after addition of flavonoids. In that case the ratios of numbers of different LPPs formed remains approximately constant, as is the ratio of total number of carbonyl and methyl groups in LPPs:

$$\frac{n_\mathrm{C}(\mathrm{LPP})}{n_\mathrm{M}(\mathrm{LPP})} = \frac{N_\mathrm{C}(\mathrm{LPP_1})n(\mathrm{LPP_1}) + \cdots + N_\mathrm{C}(\mathrm{LPP_k})n(\mathrm{LPP_k})}{N_\mathrm{M}(\mathrm{LPP_1})n(\mathrm{LPP_1}) + \cdots + N_\mathrm{M}(\mathrm{LPP_k})n(\mathrm{LPP_k})} \quad \text{(A8)}$$

$$\frac{n_C(\mathrm{LPP})}{n_M(\mathrm{LPP})} = \frac{N_\mathrm{C}(\mathrm{LPP_1}) + \cdots + N_\mathrm{C}(\mathrm{LPP_k})\dfrac{n(\mathrm{LPP_k})}{n(\mathrm{LPP_1})}}{N_\mathrm{M}(\mathrm{LPP_1}) + \cdots + N_\mathrm{M}(\mathrm{LPP_k})\dfrac{n(\mathrm{LPP_k})}{n(\mathrm{LPP_1})}} = K \approx \mathrm{const.} \tag{A9}$$

Therefore, we can express the equations (A3) and (A4) as:

$$A^0(\nu(\mathrm{C{=}O})) = \varepsilon_\mathrm{C}^0(\mathrm{DOPC}) \cdot \frac{N_\mathrm{C}(\mathrm{DOPC})}{N_\mathrm{M}(\mathrm{DOPC})} n_\mathrm{M}^0(\mathrm{DOPC}) + \varepsilon_\mathrm{C}^0(\mathrm{LPP}) \cdot K n_\mathrm{M}^0(\mathrm{LPP}) \tag{A10}$$

$$A^\mathrm{LP}\big(\nu(\mathrm{C{=}O})\big) = \varepsilon_\mathrm{C}^\mathrm{LP}(\mathrm{DOPC}) \cdot \frac{N_\mathrm{C}(\mathrm{DOPC})}{N_\mathrm{M}(\mathrm{DOPC})} n_\mathrm{M}^\mathrm{LP}(\mathrm{DOPC}) + \varepsilon_\mathrm{C}^\mathrm{LP}(\mathrm{LPP}) \cdot K n_\mathrm{M}^\mathrm{LP}(\mathrm{LPP}), \tag{A11}$$

$$A^0(\nu(\mathrm{C{=}O})) = \varepsilon_\mathrm{C}^0(\mathrm{DOPC}) \cdot n_\mathrm{M}^0(\mathrm{DOPC}) + \bar{\varepsilon}_\mathrm{C}^0(\mathrm{LPP}) \cdot n_\mathrm{M}^0(\mathrm{LPP}) \tag{A10}$$

$$A^\mathrm{LP}\big(\nu(\mathrm{C{=}O})\big) = \varepsilon_\mathrm{C}^\mathrm{LP}(\mathrm{DOPC}) \cdot n_\mathrm{M}^\mathrm{LP}(\mathrm{DOPC}) + \bar{\varepsilon}_\mathrm{C}^\mathrm{LP}(\mathrm{LPP}) \cdot n_\mathrm{M}^\mathrm{LP}(\mathrm{LPP}), \tag{A11}$$

where we introduced averaged absorption coefficient. This coefficient will be constant as long as the ratios of different LPPs remain constant.

The relative intensities *r* are

$$r^0 = \frac{A^0(\nu(\mathrm{C{=}O}))}{A^0(\nu_\mathrm{as}(\mathrm{CH_3}))}, r^{LP} = \frac{A^\mathrm{LP}(\nu(\mathrm{C{=}O}))}{A^\mathrm{LP}(\nu_\mathrm{as}(\mathrm{CH_3}))} \tag{A12}$$

or

$$r^0 = \frac{\bar{\varepsilon}_\mathrm{C}^0(\mathrm{DOPC}) \cdot n_\mathrm{M}^0(\mathrm{DOPC}) + \bar{\varepsilon}_\mathrm{C}^0(\mathrm{LPP}) \cdot n_\mathrm{M}^0(\mathrm{LPP})}{\varepsilon_\mathrm{M} \cdot n_\mathrm{M,tot}}, \tag{A13}$$

$$r^\mathrm{LP} = \frac{\bar{\varepsilon}_\mathrm{C}^\mathrm{LP}(\mathrm{DOPC}) \cdot n_\mathrm{M}^\mathrm{LP}(\mathrm{DOPC}) + \bar{\varepsilon}_\mathrm{C}^\mathrm{LP}(\mathrm{LPP}) \cdot n_\mathrm{M}^\mathrm{LP}(\mathrm{LPP})}{\varepsilon_\mathrm{M} \cdot n_\mathrm{M,tot}} \tag{A14}$$

$$r^0 = \frac{\bar{\varepsilon}_\mathrm{C}^0(\mathrm{DOPC}) \cdot x_\mathrm{M}^0(\mathrm{DOPC}) + \bar{\varepsilon}_C^0(\mathrm{LPP}) \cdot x_M^0(\mathrm{LPP})}{\varepsilon_\mathrm{M}}, \tag{A15}$$

$$r^{LP} = \frac{\bar{\varepsilon}_\mathrm{C}^\mathrm{LP}(\mathrm{DOPC}) \cdot x_\mathrm{M}^\mathrm{LP}(\mathrm{DOPC}) + \bar{\varepsilon}_\mathrm{C}^\mathrm{LP}(\mathrm{LPP}) \cdot x_\mathrm{M}^\mathrm{LP}(\mathrm{LPP})}{\varepsilon_\mathrm{M}} \tag{A16}$$

$$r^0 = \frac{\bar{\varepsilon}_\mathrm{C}^0(\mathrm{DOPC}) + \left(\bar{\varepsilon}_C^0(\mathrm{LPP}) - \bar{\varepsilon}_C^0(\mathrm{DOPC})\right) \cdot x_\mathrm{M}^\mathrm{LP}(\mathrm{LPP})}{\varepsilon_\mathrm{M}}, \tag{A17}$$

$$r^{LP} = \frac{\bar{\varepsilon}_\mathrm{C}^\mathrm{LP}(\mathrm{DOPC}) + \left(\bar{\varepsilon}_\mathrm{C}^\mathrm{LP}(\mathrm{LPP}) - \bar{\varepsilon}_\mathrm{C}^\mathrm{LP}(\mathrm{DOPC})\right) \cdot x_\mathrm{M}^\mathrm{LP}(\mathrm{LPP})}{\varepsilon_\mathrm{M}} \tag{A18}$$

where we introduce the methyl group's molar fractions as a way to quantify the amount of DOPC and LPPs in the system. We will assume that absorption coefficients do not change after lipid peroxidation, but we will allow for them to change with the addition of flavonoids in the system, as this can change the ratios of LPPs formed.

$$r_i^0 = \frac{\bar{\varepsilon}_{\mathrm{C},i}(\mathrm{DOPC}) + \Delta\,\bar{\varepsilon}_i \cdot x_{\mathrm{M},i}^0(\mathrm{LPP})}{\varepsilon_\mathrm{M}}, \tag{A19}$$

$$r_i^{LP} = \frac{\bar{\varepsilon}_{\mathrm{C},i}(\mathrm{DOPC}) + \Delta\,\bar{\varepsilon}_i \cdot x_{\mathrm{M},i}^{\mathrm{LP}}(\mathrm{LPP})}{\varepsilon_\mathrm{M}} \tag{A20}$$

The relative changes after lipid peroxidation are:

$$\rho_i = \frac{r_i^{\mathrm{LP}} - r_i^0}{r_i^0} = \frac{\Delta\,\bar{\varepsilon}_i \cdot \Delta x_{\mathrm{M},i}(\mathrm{LPP})}{\bar{\varepsilon}_{\mathrm{C},i}(\mathrm{DOPC}) + \Delta\,\bar{\varepsilon}_i \cdot x_{\mathrm{M},i}^0(\mathrm{LPP})}. \tag{A21}$$

If the fraction of LPPs before peroxidation is negligible, this equation reduces to:

$$\rho_i = \frac{\Delta\,\bar{\varepsilon}_i}{\bar{\varepsilon}_{\mathrm{C},i}(\mathrm{DOPC})} \Delta x_{\mathrm{M},i}(\mathrm{LPP}). \tag{A22}$$

If we define:

$$R_i = \frac{\rho_i - \rho_0}{\rho_0} = \frac{\frac{\Delta\,\bar{\varepsilon}_i}{\bar{\varepsilon}_{\mathrm{C},i}(\mathrm{DOPC})} \Delta x_{\mathrm{M},i}(\mathrm{LPP}) - \frac{\Delta\,\bar{\varepsilon}_0}{\bar{\varepsilon}_{\mathrm{C},0}(\mathrm{DOPC})} \Delta x_{\mathrm{M},0}(\mathrm{LPP})}{\frac{\Delta\,\bar{\varepsilon}_0}{\bar{\varepsilon}_{\mathrm{C},0}(\mathrm{DOPC})} \Delta x_{\mathrm{M},0}(\mathrm{LPP})}. \tag{A23}$$

and assume that the change in the absorption coefficients is negligible, this corresponds to:

$$R_i = \frac{\Delta x_{\mathrm{M},i}(\mathrm{LPP}) - \Delta x_{\mathrm{M},0}(\mathrm{LPP})}{\Delta x_{\mathrm{M},0}(\mathrm{LPP})}. \tag{A24}$$

From equation (A24) it is evident that with these assumptions R_i can be used to quantify inhibition.
